# Multimodal Approaches for Regenerative Stroke Therapies: Combination of Granulocyte Colony-Stimulating Factor with Bone Marrow Mesenchymal Stem Cells is Not Superior to G-CSF Alone

**DOI:** 10.3389/fnagi.2014.00130

**Published:** 2014-06-23

**Authors:** Adrian Tudor Balseanu, Ana-Maria Buga, Bogdan Catalin, Daniel-Christoph Wagner, Johannes Boltze, Ana-Maria Zagrean, Klaus Reymann, Wolf Schaebitz, Aurel Popa-Wagner

**Affiliations:** ^1^Center of Clinical and Experimental Medicine, University of Medicine and Pharmacy of Craiova, Craiova, Romania; ^2^Department of Psychiatry, University Medicine of Rostock, Rostock, Germany; ^3^Fraunhofer Institute for Cell Therapy and Immunology, Leipzig, Germany; ^4^Translational Center for Regenerative Medicine, University of Leipzig, Leipzig, Germany; ^5^Stroke and Neurovascular Regulation Laboratory, Massachusetts General Hospital and Harvard Medical School, Charlestown, MA, USA; ^6^Carol Davila University of Medicine and Pharmacy, Bucharest, Romania; ^7^German Center for Neurodegenerative Diseases (DZNE) Magdeburg in collobaration with Leibniz Institute for Neurobiology, Magdeburg, Germany; ^8^Evangelisches Krankenhaus Bielefeld gGmbH Akademisches Lehrkrankenhaus der Universität Münster, Münster, Germany

**Keywords:** aging, stroke, cell therapy, G-CSF, translational medicine, BM MSC, angiogenesis

## Abstract

Attractive therapeutic strategies to enhance post-stroke recovery of aged brains include methods of cellular therapy that can enhance the endogenous restorative mechanisms of the injured brain. Since stroke afflicts mostly the elderly, it is highly desirable to test the efficacy of cell therapy in the microenvironment of aged brains that is generally refractory to regeneration. In particular, stem cells from the bone marrow allow an autologous transplantation approach that can be translated in the near future to the clinical practice. Such a bone marrow-derived therapy includes the grafting of stem cells as well as the delayed induction of endogenous stem cell mobilization and homing by the stem cell mobilizer granulocyte colony-stimulating factor (G-CSF). We tested the hypothesis that grafting of bone marrow-derived pre-differentiated mesenchymal cells (BM-MSCs) in G-CSF-treated animals improves the long-term functional outcome in aged rodents. To this end, G-CSF alone (50 μg/kg) or in combination with a single dose (10^6^ cells) of rat BM MSCs was administered intravenously to Sprague-Dawley rats at 6 h after transient occlusion (90 min) of the middle cerebral artery. Infarct volume was measured by magnetic resonance imaging at 3 and 48 days post-stroke and additionally by immunhistochemistry at day 56. Functional recovery was tested during the entire post-stroke survival period of 56 days. Daily treatment for post-stroke aged rats with G-CSF led to a robust and consistent improvement of neurological function after 28 days. The combination therapy also led to robust angiogenesis in the formerly infarct core and beyond in the “islet of regeneration.” However, G-CSF + BM MSCs may not impact at all on the spatial reference-memory task or infarct volume and therefore did not further improve the post-stroke recovery. We suggest that in a real clinical practice involving older post-stroke patients, successful regenerative therapies would have to be carried out for a much longer time.

## Introduction

Stroke is a heavily undertreated disease demanding a vigorous search for new therapies. Attractive therapeutic strategies stimulating and finally enhancing the natural post-stroke regeneration process include methods of training such as physio- or rehabilitative therapy or methods of cellular therapy (Liepert et al., 2004; Hermann and Chopp, 2012; Honmou et al., 2012).

Studies of stroke have demonstrated an age and gender effects on incidence, functional recovery and mortality, not only in humans but also in animal models (Bergerat et al., 2011; Gokcay et al., 2011). Therefore, studies on physiologically complex organisms like rats, mice, or non-human primates are required to investigate the molecular mechanisms of aging in humans or to predict human responses to age-related diseases or the response of aged organisms to drugs. The aged rodent model offers a useful tool to investigate the molecular pathways and drugs to improve functional outcome after cerebral ischemia in preclinical studies. Over the past 10 years, suitable models for stroke in aged rats have been established. All are based on the middle cerebral artery occlusion (MCAO) (Buga et al., 2013).

To date, all monotherapeutic attempts to prevent or lessen brain damage following stroke have failed. In view of our findings that stroke impacts a wide range of systems in an age-dependent manner, from CNS physiology to CNS regeneration and plasticity (Popa-Wagner et al., 2011; Buga et al., 2013, 2014), the failure of therapies aimed at only a single target system is perhaps inevitable. We hypothesize that a multi-stage and multimodal treatment in aged animals may be more likely to produce positive results.

The endogenous approach [granulocyte colony-stimulating factor (G-CSF)-induced stem cell mobilization] has been shown to enhance post-stroke recovery and to reduce infarct volumes even when treatment was delayed for several days after stroke onset (Schneider et al., 2005; Lu and Xiao, 2006; Schäbitz and Schneider, 2007). G-CSF treatment was therefore translated into human stroke where the safety of several doses could be established (Schäbitz et al., 2008; England et al., 2012; Ringelstein et al., 2013).

Cellular therapy can enhance the endogenous restorative mechanisms of the injured brain by supporting processes of neovascularization, neurogenesis, and neural reorganization (Chen et al., 2005; Crigler et al., 2006; Hayase et al., 2009; Bao et al., 2011; Lim et al., 2011; Hsieh et al., 2013). Several studies showed that grafting of BM MSCs in the peripheral circulation improved functional neurological outcome and reduced infarct volume (Honmou et al., 2012). Most of these studies used BM MSCs but feasibility and safety in clinical trials was also shown for the bone marrow mononuclear cells (BM MNCs) (Hermann and Chopp, 2012; Moniche et al., 2012). A conclusive result on optimal timing and dosing is, however, still missing.

In a previous work, we have shown that application of G-CSF shortly after stroke in aged rats increases neurogenesis and improves some of the behavioral indices (Popa-Wagner et al., 2010). Therefore, we reasoned that the efficiency of the bone marrow-derived-cell therapy may be increased by simultaneous application of G-CSF. In particular, we tested the hypothesis that grafting of pre-differentiated mesenchymal cells in G-CSF-treated animals improves long-term functional outcome in aged rodents.

## Materials and Methods

### Animals and experimental design

The subjects of these experiments were aged male Sprague-Dawley rats (*N* = 80; 18–20 months of age; 520–600 g) kept under standard laboratory conditions with free access to food and water. The numbers reported in the results refer to the number of animals that survived the surgery and completed the 8-week testing period. All experiments were approved by the Animal Experimentation Ethics Board of the State of Mecklenburg-Vorpommern as meeting the ethical requirements of the German National Act on the Use of Experimental Animals (approval no LALLF M-V/TSD/7221.3-1.1-040/10) and by the Institutional Animal Care and Use Committee of the Medical University of Craiova.

### Randomization

A scientist was in charge of randomization by (i) group assignment, (ii) surgery assignment; and (iii) treatment assignment.

### Behavioral testing

To evaluate changes in neurological function associated with ischemia, the rats were subjected to a variety of somatosensory, motor, learning, and memory tests before and after surgery. All testing was performed from 9 to 11 a.m. Results obtained before surgery were used to define 100% functionality for each animal on each test, and functional recovery was expressed as percent recovery relative to the pre-surgery baseline.

### Rotating pole

The rotating pole task assesses coordination and sensorimotor function in the MCAO model. Each rat was tested for its ability to cross a rotating (6 rpm) horizontal rod. The score assessment was done as previously described by our group (Buchhold et al., 2007). Briefly, the time taken for the rat to traverse the rotating cylinder and join a group of rats visible at the finish line was measured. The score assessment was twofold: (i) time (seconds) required to traverse the rotating cylinder and (ii) the score as follows: **0** – rat falls immediately (onto a soft surface); **1** – rat does not walk forward, but stays on the rotarod; **2** – rat walks, but falls before reaching the goal; **3** – rat traverses the rod successfully, but the limbs are used asymmetrically; **4** – the left hindlimb is used less than 50% of the time taken to traverse the rod; **5** – the rat successfully traverses the rod, but with some difficulties; **6** – no mistakes, symmetric movements.

### Asymmetric sensorimotor deficit: Adhesive tape removal test

We assessed the asymmetry of sensorimotor deficit of the forelimbs induced by unilateral MCAO by the adhesive tape removal test. In short, sticky patches were applied on the distal hairless parts of the forelimbs and the removal time from both limbs was measured. Three trials were done separately for each limb and the means of the values were noted. If the animal did not remove the tape within 180 s, the timer was stopped. Results are given as time needed to remove the adhesive tape from one forelimb divided by the sum of time needed to remove it from both forelimbs (Popa-Wagner et al., 2010).

### Morris water maze

The Morris water maze task was used to assess spatial learning and memory. One week before surgery, aged rats were trained to find a submerged platform in a large (180 cm-diameter) pool filled to within 20 cm of the upper edge with water maintained at 26°C. The pool was divided into four compass quadrants (north, south, east, and west). Several visual stimuli were placed in each of the four quadrants. For the acquisition of spatial learning, each animal underwent a block of four trials per day for 7 days. Before the first trial, the rat was placed on the hidden platform for 30 s by the investigator. Each trial consisted of placing the rat in the water at one of the randomly selected four starting locations around the pool perimeter. Each rat was allowed a maximum of 60 s to find the hidden platform and remain on it for 30 s. If a rat failed to find the platform within 60 s, the rat was placed on the platform for 30 s by the investigator. The time and distance required to find the hidden platform during these four acquisition trials were averaged (Tottori et al., 2002). The swim path was recorded by an image analysis system (VideoMot2, TSE, Bad Homburg, Germany) that computed path length and percentage of time spent in each of the four quadrants. Functional restoration of spatial learning and memory was estimated by weekly testing after MCAO and in total for 8 weeks.

### Preparation of RAT bone marrow-derived mesenchymal cells (rBM-MSC)

Bone marrow was collected from 40 Sprague-Dawley (5 weeks old; Harlan-Winkelmann, Borchen, Germany). Both femurs were aseptically removed and placed in alpha-MEM (Biochrom, Germany) supplemented with 20% fetal bovine serum (FBS, Biochrom, Germany), 1% penicillin/streptomycin (Biochrom, Germany), and 2 mM HEPES (Biochrom, Germany), to which we refer as standard medium. The bone marrow was flushed out with 5 ml standard medium using a 10 ml-syringe and a 23-gage needle and the BM MSC was isolated and cryopreserved as previously described (Sauerzweig et al., 2009). The cells were counted using a Neubauer-hemocytometer and cultured with a density of 2 × 10^6^ whole marrow cells per square centimeter in standard medium with 37°C, 5% CO_2_, and 92% relative humidity (RH). After 3 days, non-adherent cells were removed by flushing with 10 ml PBS 0.1 M and the standard medium was replaced. The attached cells were cultured until 80% of confluence was reached. After trypsinization, cells were dissolved in standard medium supplemented with 10% DMSO (Sigma-Aldrich, Germany) and apportioned for cryo-preservation. Immunophenotyping of rBM-MSCs was performed with antibodies against rat antigens CD29, CD45, and CD90.

### Preparation of human BM MSCs (hBM MSCs)

Mesenchymal stem cells (MSCs) harvested and cultured from normal human bone marrow were purchased from Lonza Research (Cologne, Germany) and cultivated according to manufacturer’s protocol under the same conditions as rat cells. The phenotypes of hBM MSCs used in this study were positive for CD105 and CD166. Cells tested negative for CD14, CD34, and CD45. Cells were tested for purity by flow cytometry and for their ability to differentiate into osteogenic, chondrogenic, and adipogenic lineages by Lonza.

### Induction of stroke

Eighteen hours prior to surgery, rats were deprived of food to minimize variability in ischemic damage that can result from varying plasma glucose levels. Water remained available at all times. In all cases, surgery was performed between 9:00 and 13:00 hours.

Cerebral infarction was induced by transcranial interruption of blood flow by transiently lifting the middle cerebral artery (MCA) with a tungsten hook as previously described (Popa-Wagner et al., 2010). Throughout surgery, anesthesia was maintained by spontaneous inhalation of 1–1.5% halothane in a mixture of 75% nitrous oxide and 25% oxygen. Body temperature was maintained at 37°C by a Homeothermic Blanket System (Harvard Apparatus). The local changes in blood flow were monitored using a laser Doppler device (Perimed, Stockholm, Sweden). A decrease in the laser Doppler signal to <20% of control values was considered to indicate successful MCA occlusion. After 90 min, the tungsten hook was released and the common carotid arteries were re-opened. Surgery was performed under antiseptic conditions to minimize the risk of infection. Subsequent to survival times of 56 days, the rats were deeply anesthetized with 2.5% halothane in 75% nitrous oxide and 25% oxygen, and perfused with neutral buffered saline followed by buffered 4% freshly depolymerized paraformaldehyde. The brain was removed, post-fixed in 4% buffered paraformaldehyde for 24 h, cryoprotected in 15% glycerol prepared in 10 mmol/l phosphate buffered saline, flash-frozen in isopentane, and stored at −70°C until sectioning.

### Treatments

**Figure 1:**
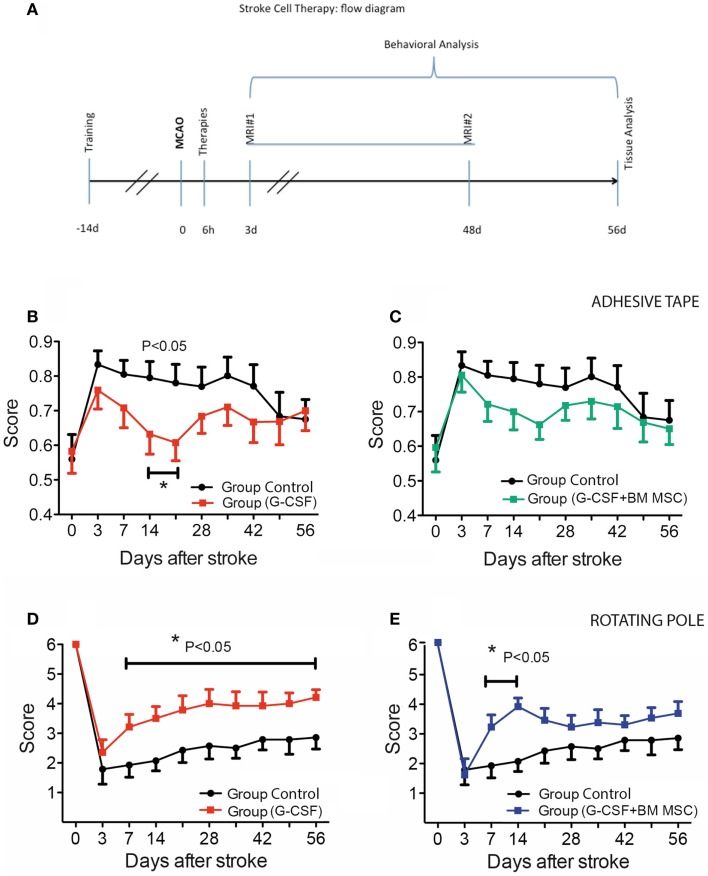
**Experimental design and time course of cutaneous sensitivity and sensorimotor integration recovery after stroke therapy**. **(A)** Schematic overview of the experimental design. **(B,C)** Time course of cutaneous sensitivity and sensorimotor integration recovery after stroke therapy by the adhesive tape removal test. By day 3, post-stroke animals started recuperation and reached significant recovery of function by day 14 in the G-CSF group [**(B)**, filled red squares] as compared to the control group [**(B)**, filled black circles]. The combination of G-CSF and BM MSC showed no significant improvement of recuperation of sensorimotor function [**(C)**, filled green squares] vs controls [**(C)**, filled black circles]. **(D,E)** Functional recovery on the rotating beam. Control rats began improvement and recovered to 47% by day 56 [**(D)**, **(E)** filled black circles]. Of the treated groups, best recovery of the bilateral sensorimotor coordination was shown in G-CSF alone that reached 72% of the pre-surgery value [**(D)**, red squares] followed by G-CSF + BM MSC [58%; **(E)**, blue squares]. Data are given as mean ± SEM.

Animals were randomly assigned to three groups as follows: (i) G-CSF group (*N* = 20) received daily injections of 50 μg/kg BW and in total for 28 days; (ii) G-CSF + BM MSCs (*N* = 20) group received daily injections of G-CSF at 50 μg/kg BW and in total for 28 days and a single dose of BM MSCs (1 × 10^6^/kg BW) given intravenously; and (iii) control group (*N* = 20) was given daily the vehicle (5% glucose) for 28 days. Combination treatment was done at 6 h post-stroke (Figure 1, upper panel). To investigate the localization of injected cells, a separate group of aged animals was injected with mesenchymal cells of human origin (hBM-MSCs). Although hBM-MSCs are poorly immunogenic (Chamberlain et al., 2007) and the rats survived just 4 days after administration, to prevent graft rejection the animals were given cyclosporine A (s.c., Sandimmun, Novartis, 10 mg/kg) diluted in Chremophor EL (Sigma).

### BrdU labeling

To label newly generated cells, rats were given bromodeoxyuridine (BrdU; 50 mg/kg body weight, i.p.; Sigma), daily in the first week post-stroke, and every other day in the following weeks post-stroke, for a total period of 4 weeks.

### Magnetic resonance imaging

Magnetic resonance imaging (MRI) was used to visualize the infarct volume for both groups at day 3 and at day 48 after stroke. MRI measurements were performed on a 7-T Bruker ClinScan magnet with a 20 cm inner bore, capable of 290 mT/m in 250 μs (Bruker BioSpin MRI, Ettlingen, Germany). Images were received by a 2 × 2 phased array RF coil, designed specifically for rat brain studies that was placed directly on the skull. The animals were anesthetized during imaging to minimize discomfort. Respiratory rate was monitored, and isoflurane concentrations were varied between 1.5 and 2.0% to keep the respiratory rate between 35 and 45/min. After positioning the animal’s head, quantitative T2 measurements were performed with a multislice spin-echo sequence with 25 slices of 0.7 mm thickness and a matrix size 640 × 640 pixels, a repetition time (TR) of 4330 ms, and an echo time (TE) of 45 ms.

### Lesion measurement using MIPAV software

T2WI lesion volumes were determined using the image processing software Medical Image Processing, Analysis and Visualization (MIPAV, version 3.0, National Institutes of Health, Bethesda, MD, USA). After optimal adjustment of contrast, the edge of the lesion was traced manually on each of the 25 coronal slices, which completely covered the MCA territory in all animals. The areas of hyperintensity were then summed and multiplied by the slice thickness to calculate lesion volumes.

### Determination of infarct volume by immunohistostaining

To assess the size of the infarct induced by focal ischemia, we introduced several years ago NeuN immunostaining (Badan et al., 2003). Every 20th free-floating section of 25 μm was immunostained for NeuN to cover the entire infarcted volume, which was then calculated as the sum of the partial areas using the MicroBrightField (Colchester, VT, USA) system. Integration of the resulting partial volumes (partial areas × no. of sections × section thickness × section intervals) yielded the total volume of the ipsilateral hemisphere along with the total volume of the infarct.

### Immunofluorescence

Cryostat, free-floating sections of 25 μm were fixed in 4% paraformaldehyde for 15 min and then washed extensively with PBS and processed using an automatic staining machine for floating sections Tingomat 501^1^. After incubation in 50% formamide/2× SSC for 2 h at 60°C, sections were washed again, first in 2× SSC and then in 10× PBS. After denaturization in 2 N HCL at 37°C for 40 min, sections were made neutral by adding 0.1 M borate buffer (pH 8.5). Thereafter, sections were incubated with guinea pig anti-doublecortin (DCX; Millipore, Germany) overnight at 4°C followed by secondary biotinylated antibodies (Dianova, Hamburg, Germany) and visualized with streptavidin Alexa 488 (Life Technologies, Karslruhe, Germany). Finally, sections were incubated with rat anti-BrdU antibody (1:2000, AbD Serotec, Puchheim, Germany). BrdU-positive cells were visualized by incubating with Cy3-conjugated donkey anti-rat IgG (H + L) (1:3000). For phenotyping in the periinfarcted area and beyond the fibrotic scar, sections were triple-immunolabeled with rabbit anti-laminin-specific antibodies (1:5000, Sigma, Munich), mouse anti-NeuN antibodies (1:500, Millipore, Germany), mouse anti-RECA (1:1000, Millipore, Germany), and rat anti-BrdU-specific antibodies (1:3000; Serotec, UK). The antigen–antibody complexes were visualized with donkey anti-mouse Cy3-conjugated antibodies (1:2000), donkey anti-rabbit Cy2-conjugated antibodies (1:3000), and donkey anti-rat Cy5-conjugated antibodies (1:2000), respectively.

For phenotyping of transplanted cells, the tissue was incubated with a rabbit anti-mouse NeuN (1:1000, Novus Biologicals, UK) or mouse anti-human CD166 (1:1000, antibodies-on-line, Aachen, Germany) or mouse anti-human nuclei (ABIN361360 antibodies-on-line, Aachen, Germany) or mouse anti-human CD105 antibodies at 4°C overnight. At the next day, sections were rinsed with PBS and incubated with Alexa Fluor^®^ 568 goat anti-rabbit IgG or Alexa Fluor^®^ 594 goat anti-mouse IgG for CD105. Finally, the signal was amplified utilizing an anti-mouse polymer-based secondary detection system (Histofine polymer-HRP, Nichirei, Japan) diluted 1:10 in PBS containing 1% normal goat serum and 0.3% Tween 20. After washing in PBS, sections were stained with tyramide-FITC. After final rinsing, sections were brought to Superfrost Plus slides and mounted using PVA/DABCO-containing medium. To visualize nuclei, some sections were counterstained with DAPI.

### Quantitation of microvascular density

Microvascular density was quantitated using the “hot spot” analysis covering 30% of the infarcted area. Briefly, hot-spots, i.e., regions with a high density of CD105-positive microvessels in humans and CD31-capillaries in rats, were identified using a 40× objective and were then counted using 20× objective, corresponding to a microscopic field of 0.7386 mm^2^. Counting was done by two independent observers and the results are expressed as means ± SD (Buga et al., 2014).

### Microscopy

For light microscopy, a Nikon Eclipse (Nikon, Duesseldorf, Germany) was used. Confocal microscopy images were acquired using a Zeiss LSM710 laser-scanning confocal system with spectral detection capabilities, and Zen 2010 software version 6.0 (Carl Zeiss Microscopy GmbH, Jena, Germany) was used for image acquisition and analysis. Excitation light was provided by 488, 543, and 634 nm laser lines; fluorescence emission was detected at 500–530 nm for FITC (green), 550–600 nm for rhodamin (red) and 650–710 nm for Cy5 (blue) in separate tracks, using a confocal aperture of 1 Airy unit. Some of the images were acquired as *z*-stacks and 3D reconstruction was performed.

### Statistical analysis

The main effects of treatment and time as well as interactions of the two factors, on recovery, were evaluated using a mixed-model analysis of variance (MANOVA) for each measure, with treatment as between-subjects variables and time as a within-subject variable. The non-parametric data analysis was conducted using the Kruskal–Wallis test that is designed for multiple independent measures followed by a Bonferroni correction for alpha errors (SPSS Inc., Chicago, IL, USA). A *p* value of <0.05 was considered to be statistically significant. Water maze analysis was done using the Wilcoxon rank sum test.

## Results

### General observations

To facilitate feeding in aged animals during the first 3 days post-stroke, we fed them with moistened, soft pellets. Following infarction, all rats had diminished performance on the first 3 days post-surgery, which was at least partially attributable to the surgical procedure itself. The mortality rate was almost identical, 10% for each group. All the above mentioned animals died between day 3 and day 10 post-stroke.

### The effect of G-CSF and its combination with BM MSCs on functional recovery in post-stroke aged rats

The adhesive tape removal test probes for differences between forelimbs in cutaneous sensitivity and sensorimotor integration after stroke. As compared to pre-operative, trained animals, rMCAo animals demonstrated a marked difference in post-operative performance for the left (affected) forelimb. By day 3, post-stroke animals started recuperation and reached significant recovery of function by day 14 in the G-CSF group only (Figure 1B, filled red squares). The combination between G-CSF and BM MSC showed no additional or showed a similar although non-significant trend improvement of recuperation of sensorimotor function (Figure 1C, filled green squares).

After an abrupt decline in performance on the rotarod on day 3 post-stroke, control rats began improvement and recovered to 47% by day 56 (Figure 1D, filled black circles). Of the treated groups, the best recovery was shown by group treated with G-CSF alone that reached 72% of the pre-surgery value (baseline) (Figure 1D, red squares) followed by the group treated with G-CSF + BM MSC that reached 58% of the pre-stroke value (Figure 1E, blue squares).

**Figure 2:**
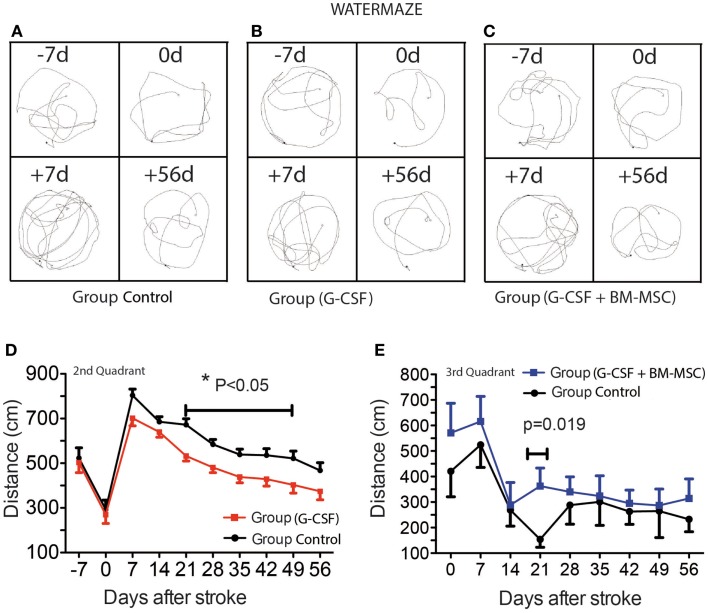
**Time course of post-stroke recovery of learning and (spatial) memory by water maze**. Representative swim paths are shown in **(A–C)** and included the start of training (−7d), the pre-surgery path pattern (0d), first testing after stroke (+7d), and the final testing (+56d). The best recovery was seen for the G-CSF group that showed significant improvement of spatial reference-memory between days 21 and 49 in the second quadrant [**(D)**; *p* = 0.05]. However, in the third quadrant, the performance was temporarily improved between days 14 and 28 in the group treated with G-CSF + BM MSC as compared to the control group [**(E)**; *p* = 0.019]. Data are given as mean ± SEM.

Representative swim paths are shown in Figures 2A–C and included the start of training (−7d), the pre-surgery path pattern (0d), first testing after stroke (+7d), and the final testing (+56d). Over the pre-stroke training period of 7 days, rats learned to locate and climb onto the hidden platform and performance improved significantly during this time. In all groups, the path became shorter as the training sessions progressed (Figures 2A–C). Because of the skull injury, we avoided testing the animals in the first week post-stroke.

As previously shown, aged rats need more time to recover behaviorally after stroke than young animals (Lindner, 1997; Tottori et al., 2002; Lindner et al., 2003; Buchhold et al., 2007). Consequently, the path length required to reach the platform in the third quadrant reached a maximum by day 7 post-stroke. After 7 days, the animals began recovering in this test. The best recovery was seen for the G-CSF group that showed significant improvement of spatial reference-memory between days 21 and 49 in the second quadrant (Figure 2D). However, in the third quadrant, the performance was temporarily improved between days 14 and 28 in the group treated with G-CSF + BM MSC as compared to the control group (Figure 2E).

### The effect of cell therapy on the infarct volume

**Figure 3:**
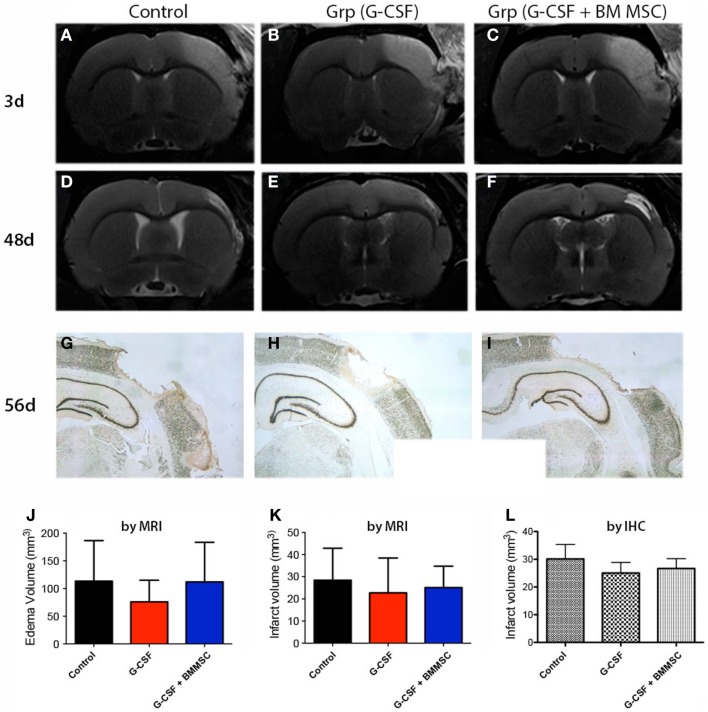
**Edema and stroke volumes by MRI and NeuN immunohistochemistry**. **(A–C)** Perilesional brain edema at day 3 post-stroke, as defined by the region of T2 hyperintensity **(A)** was not significantly reduced by any treatment **(J)**. **(D–F)** The second MRI done at day 48 **(B)** post-stroke revealed much smaller infarcts. **(G–I)** By immunohistochemistry at day 58, the infarct volumes for controls **(G)**, G-CSF alone **(H)**, and combination treatment **(I)** were largely similar to those measured by MRI at day 48. The infarct volume after 7–8 weeks post-stroke was not significantly different among the groups **(K,L)**.

Representative MRI data for the two rats closest to the mean for each group are shown in Figures 3A–F. Cortical lesion and brain edema at day 3 post-stroke, as defined by the region of T2 hyperintensity, was not significantly reduced by any treatment (Figures 3A–C). The G-CSF group showed, however, less edema as compared with the combination treatments or controls (Figure 3J, red bar). The second MRI done at day 48 post-stroke revealed much smaller infarcts suggesting that brain edema has had a large contribution to the T2 hyperintensity in aged animals (Figures 3D–F).

Immunohistochemical staining of the infarct area at day 56 using an anti-NeuN antibody (Figures 3G–I) showed that the infarct volumes were largely similar to those measured by MRI at day 48, indicating that the infarct volume had stabilized by day 48. Consequently, the infarct volume as measured by MRI (Figure 3K) or immunohistochemistry (Figure 3L) was not significantly different between the groups at day 48 post-stroke.

### Localization of human BM MSCs by phenotyping

**Figure 4:**
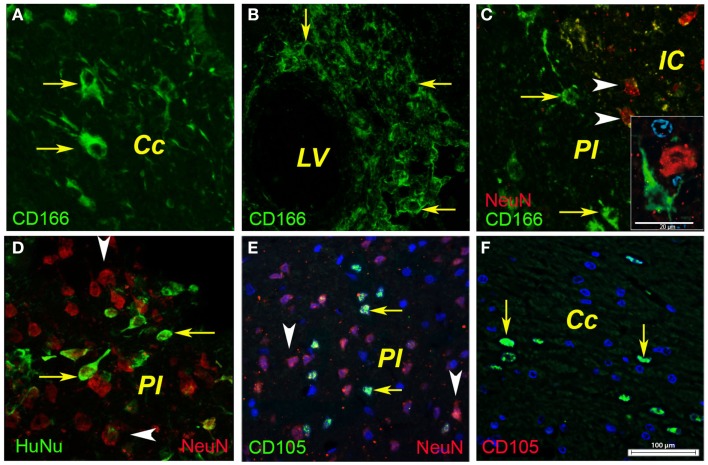
**Phenotyping of human BMSCs**. In the ipsilateral hemisphere, the injected human BMSCs were localized in the corpus callosum as shown for CD166-positive cells [**(A)**, arrows] and CD105-positive cells [**(F)**, arrows]. In our model, the cells most likely entered the injured brain via the lateral ventricle as shown by the CD166-positive cells **(B)**. A fraction (about 1%) of the injected CD166- and CD105-positive cells reached the infarcted area [**(C,E)**, arrows] where they were intermingled with surviving or degenerating neuronal nuclei [**(C)**, arrowheads]. Noteworthy was also the presence of immunopositivity for human nuclei [**(D)**, arrows] that were dispersed between the rat nuclei in the infarcted area [**(D)**, arrowheads]. Cc, corpus callosum; IC, infarct core; LV, lateral ventricle; PI, periinfract.

In the ipsilateral hemisphere, the injected human BM MSCs were detected in the corpus callosum as shown for CD166-positive cells (Figure 4A, arrows) and CD105-positive cells (Figure 4F, arrows). In our model the cell probably entered the injured brain via the lateral ventricle as shown by the CD166-positive cells (Figure 4B). A fraction (about 1%) of the injected CD166- and CD105-positive cells reached the infarcted area (Figures 4C,E, arrows) where they were intermingled with surviving or degenerating neuronal nuclei (Figure 4C, arrowheads). Noteworthy was also the presence of immunopositivity for human nuclei (Figure 4D, arrows) that were dispersed between the rat nuclei in the infarcted area (Figure 4D, arrowheads).

### The combined treatment did not enhance neurogenesis in the subventricular zone

**Figure 5:**
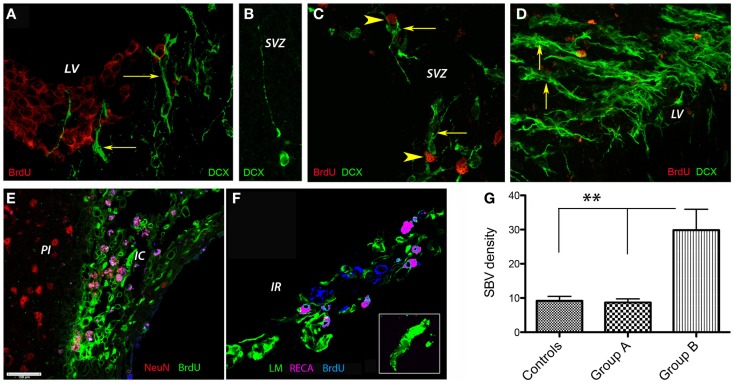
**Post-stroke neurogenesis and angiogenesis**. At 8 weeks post-stroke, none of the DCX^+^ cells in the SVZ of control animals co-localized with BrdU-labeled nuclei. Instead, the BrdU-positive nuclei were distributed mainly in the “pinwheel” architecture of the ventricular epithelium **(A)**. The DCX^+^ cells occupied an adjacent, distinct position [**(A)**, arrows]. Some of the DCX^+^ migrated away from the ventricular wall **(B)**. We noted vigorous neurogenesis with many DCX^+^ (arrows) co-localizing with BrdU nuclei in the G-CSF-treated animals [**(C)**; arrowheads] and animals treated with G-CSF + BM MSC [**(D)**, arrows]. **(E–G)** Post-stroke angiogenesis. In regions adjacent to the infarct scar, we found numerous BrdU^+^ nuclei in the endothelium of newly formed blood vessels in the formerly infarct core [**(E)**, green]. The border to the healthy brain region was abruptly demarcated to the left by NeuN-positive nuclei [**(E)**, red]. Beyond the formerly infarct core, we noted vigorous sprouting angiogenesis as evidenced by RECA/BrdU double positive blood vessels [**(F)**, violet] as well as numerous BrdU^+^ nuclei in the newly formed endothelium [**(F)**, blue] and reconstruction of the basal lamina [**(F)**, green] during the resolution phase of angiogenesis. By number of laminin/BrdU co-localizations, the density of the newly formed blood vessels was significantly higher (threefold, *p* = 0.01) in the brains of animals treated with the combination G-CSF + BM MSC as compared to controls and G-CSF alone **(G)**. Cc, corpus callosum; IC, infarct core; IR, islet of regeneration; LV, lateral ventricle; PI, periinfract.

Next, we investigated the presence of the early neuronal marker doublecortin by immunofluorescence in the lateral ventricle region. To this end, the proliferating cells were labeled by injecting animals with BrdU. To our surprise, at day 56 post-stroke none of the DCX^+^ cells in the SVZ of control animals co-localized with BrdU-labeled nuclei. Instead, the BrdU-positive nuclei were distributed mainly in the “pinwheel” architecture of the ventricular epithelium (Liebner et al., 2008; Gajera et al., 2010) (Figure 5A). The DCX^+^ cells occupied an adjacent, distinct position (Figure 5A, arrows). Some of the DCX^+^ migrated away from the ventricular wall (Figure 5B). In agreement with the previous results, we noted vigorous neurogenesis with many DCX^+^ (arrows) co-localizing with BrdU nuclei in the G-CSF-treated animals (Figure 5C; arrowheads) and animals treated with G-CSF + BM MSC (Figure 5D, arrows).

### Increased microvascular density in the G-CSF + BM MSC group

In regions adjacent to the infarct scar, we found numerous BrdU^+^ nuclei in the endothelium of newly formed blood vessels in the formerly infarct core (Figure 5E, green). The border to the healthy brain region was abruptly demarcated to the left by NeuN-positive nuclei (Figure 5E, NeuN shown in red). Beyond the formerly infarct core, we noted vigorous sprouting angiogenesis as evidenced by RECA/BrdU double positive blood vessels (Figure 5F, violet) as well as numerous BrdU^+^ nuclei in the newly formed endothelium (Figure 5F, blue) and reconstruction of the basal lamina (Figure 5F, green) during the resolution phase of angiogenesis. By number of laminin/BrdU co-localizations, the density of the newly formed blood vessels was significantly higher (threefold, *p* = 0.01) in the brains of animals treated with the combination G-CSF + BM MSC as compared to controls and G-CSF alone (Figure 5G).

## Discussion

The aged post-stroke brain microenvironment is refractory to regeneration (Conover and Shook, 2011; Popa-Wagner et al., 2011). Bone marrow-derived cell therapy includes the grafting of stem cells as well as the delayed induction of endogenous stem cell mobilization and homing by the stem cell mobilizer G-CSF (Schäbitz and Schneider, 2007; Schäbitz et al., 2008). In this study, we tested the hypothesis that such a combination is superior to G-CSF alone. We found that the aged brain environment is permissive for the migration of human BM MSC toward the lesion site. Further, the combination therapy increased neurogenesis in the subventricular zone and improved significantly recuperation and microvessel density in the formerly infarct core and beyond. However, neither G-CSF nor the combination was efficient in reducing the mortality rate and the infarct volume. These findings are consistent with previous reports using cellular post-stroke therapies (Chen et al., 2000, 2004; Zhao et al., 2002; Popa-Wagner et al., 2010) or human adult bone marrow-derived somatic cells (Braun et al., 2012).

Different routes of MSC administration have been used to treat damaged ischemic brain tissue. Of these, the intravenous infusion of BM MSCs might be a feasible and safe mode for treatment of stroke patients (Chen et al., 2003). Some other studies have shown that, on the contrary, intrathecal delivery by lumbar puncture may be a more efficient approach for the BM MSCs treatment of stroke (Lim et al., 2011).

Mesenchymal stem cells exhibit intrinsic homing properties to sites of injury, inflammation, and hypoxia (Orlic et al., 2001; Hofstetter et al., 2002; Mahmood et al., 2003; Spaeth et al., 2008) that can be used for targeted delivery of therapeutic factors. In a mouse model of glioblastoma, a large number of cells migrated toward the tumor along the corpus callosum (Menon et al., 2009). Our study shows that the aged rat brain environment still support this migratory pathway in the ischemic brain as suggested by the presence of several marker of human MSCs, CD166, and CD105 in the corpus callosum and periinfarcted area.

Cerebral ischemia can cause both physical disability and prolonged spatial memory disturbance in rodents, a phenomenon aggravated by advanced age (Buchhold et al., 2007; DiNapoli et al., 2008; Bingham et al., 2012). Improved functional recovery including performance of a skilled forelimb reaching task following transplantation of MSC of human or rat origin (Hayase et al., 2009; Bao et al., 2011; Jiang et al., 2012) or cognitive restoration estimated by Morris water maze following transplantation of MSC rat origin has also been reported (Pavlichenko et al., 2008).

Intravenous transplantation of MSCs led to motor function improvement as assessed by fMRI (Suzuki et al., 2013). In a previous study, we have shown that post-stroke G-CSF treatment of aged animals improved survival and had a beneficial effect on functional outcome in somatosensory, motor, learning, and memory tests. However, this beneficial effect was not lasting but overlapped with the G-CSF treatment period (Popa-Wagner et al., 2010). In the present study, the combination of G-CSF and BM MSC in aged rats showed, surprisingly, no additional improvement in recuperation of the sensory function (adhesive tape) although recuperation of more complex motor (rotating pole) and spatial reference-memory tasks was improved both by G-CSF and the combination. Our findings are in agreement with earlier studies showing no improvement of clinical symptoms after hMSC transplantation in a mouse model of stroke (Steiner et al., 2012).

Stroke in young animals promotes cell proliferation in the SVZ and increases the number of immature neurons (Arvidsson et al., 2002; Parent et al., 2002). Previously, we have shown that G-CSF has increased neurogenesis by day 28 in the post-stroke aged brain (Popa-Wagner et al., 2010). In the present study, the animals survived for 60 days. At day 56, neurogenesis still persisted in the ipsilateral SVZ of the aged post-stroke control brains. In control brains, the DCX^+^ cells occupied an adjacent, distinct position while some of the DCX^+^ migrated away from the ventricular wall. In agreement with previous results, we noted vigorous neurogenesis and many DCX^+^ co-localized with BrdU nuclei in the SVZ of G-CSF-treated animals.

Reports on the differentiation of transplanted cells yielded conflicting results. Earlier studies have shown that MSCs injected into the lateral ventricle of neonatal mice persistently engraft and migrate throughout the brain and adopt an astrocyte-, oligodendrocyte- (Zhao et al., 2002; Lu et al., 2008), or endothelial cell-like phenotypes (ECs) some of them being incorporated into newly formed brain vessels (Hess et al., 2002; Zhang and Harder, 2002; Foteinos et al., 2008). Occasionally, a neuronal phenotype has been reported *in vivo* (Kopen et al., 1999; Brazelton et al., 2000; Priller et al., 2001) at the site of injury (Ankeny et al., 2004; Lu et al., 2005) and *in vitro* (Deng et al., 2006). Committed neural progenitor cells, NS-MSCs, produced *in vitro* from rat and human MSCs, differentiated into neuronal cells after transplantation and became immunoreactive to various neurotransmitter-related markers within the host tissue.

In our model, the MSC reached the periinfarcted region after 4 days. However, 56 days after the administration of BM MSCs, the proliferation marker BrdU had been incorporated preferentially in the “pinwheel” architecture of the ventricular epithelium (Liebner et al., 2008; Gajera et al., 2010) and blood vessel in the formerly infarct core while the DCX^+^ cells occupied an adjacent, distinct position in the SVZ, and were not detectable at all in the lesioned area. Since BrdU was administered for the first 14 days after stroke, we concluded that at 2 months post-stroke DCX^+^ cells with BrdU nuclei did not survive strongly suggesting that the early neuronal progenitors did not survive in the hostile environment of the post-stroke aged brain. Instead, in animals treated with the combination of G-CSF and BM MSCs, we noted an increased number of newly formed blood vessels in the formerly infarct core and the region beyond it which we dubbed the “islet of regeneration” (Buga et al., 2014). These results strongly suggest that the BM MSC promoted angiogenesis rather neurogenesis in the lesioned area (Bronckaers et al., 2014). Indeed, previous studies have shown that delayed intracerebral injection of h BM MSCs modified the cerebral microvasculature after transient ischemia (Moisan et al., 2012) and improved the cerebral blood flow (CBF) (Jiang et al., 2012).

The current understanding of mechanisms underlying stroke treatment with bone marrow-derived cells is that homing of cells into the infarcted brain may cause trophic support and hereby enhanced post-stroke recovery. Transplanted NSCs may promote recovery also without differentiating to neurons and even without long-term survival through several other mechanisms, e.g., neurogenesis (Tfilin et al., 2010), modulation of inflammation (Lee et al., 2008; Horie et al., 2011), neuroprotection (Bacigaluppi et al., 2009), and stimulation of angiogenesis (Bronckaers et al., 2014) and brain plasticity (Liu et al., 2008; Andres et al., 2011). Delayed and persistent functional improvement induced by intracerebral NSC transplantation after stroke, without cell survival and tissue replacement at 6 months after the insult, was recently documented by electrophysiology, fMRI, and behavioral testing (Ramos-Cabrer et al., 2010).

Therapeutic effects mediated by cell transplantation are likely related to the secretion of growth factors and cytokines. Mechanistically, studies done on young subjects using human umbilical cord blood-derived MSCs or bone marrow-derived cell treatment for stroke suggest that homing of cells into the infarcted brain may cause trophic support for host neurons and hereby enhanced post-stroke recovery. Indeed, MSCs enriched from adult human umbilical cord and bone marrow have demonstrated therapeutic efficacy for treatment of stroke in a rat model, presumably by an increased expression of cytokines CXCL2 and CXCL5 and growth factors BDNF, NT-3, FGF9, HBEGF, and VEGF in the ischemic brain that was accompanied by reduction of the infarct volume, increased neurogenesis, and improved neurological function (Chen et al., 2005; Crigler et al., 2006; Hayase et al., 2009; Bao et al., 2011; Lim et al., 2011; Hsieh et al., 2013). However, our combination therapy did not further enhanced neurogenesis in the SVZ of aged animals.

Following stroke, angiogenesis, which is defined as formation of microvessels from existing microvessels, is enhanced in the periinfarcted region as compared to the contralateral unlesioned cortex (Wang et al., 2005; Kilic et al., 2006; Zechariah et al., 2013). Angiogenesis has multiple beneficial roles in the post-ischemic brain, contributing to (i) the stabilization of brain hemodynamics, thus preventing subsequent stroke events, (ii) the prevention of neuronal degeneration via release of neurotrophic growth factors, (iii) the removal of dead cells from the tissue by blood-derived macrophages that find access to the ischemic lesion, and (iv) the attraction of neural precursor cells that again release growth factors (Slevin et al., 2006; Hermann and Zechariah, 2009).

The aged brain is particularly refractant to growth phenomena after injuries. We and others have shown that potential mechanisms for self-repair also operate in the post-ischemic aged brain. The major factors involving the loss of regenerative capacity in the aged brain are an age-related decrease in neurogenesis and an increased inflammatory response to stroke that impairs regeneration and migration of neuronal precursors toward the ischemic lesion that is most evident in the aged brains (Popa-Wagner et al., 2011). Although we found that the aged brain environment is permissive for the migration of BM MSCs, the number of transplanted cells that have reached the periinfarcted region was too small to have any relevance for tissue and functional recovery.

## Conclusion

Daily treatment of post-stroke aged rats with 50 μg/kg G-CSF for a longer time (28 days) led to a robust and consistent improvement of several neurological functions. The combination therapy of G-CSF and BM MSCs led to robust angiogenesis in the formerly infarct core and beyond in the “islet of regeneration.” However, the combination therapy with G-CSF and BM MSCs was not superior to G-CSF alone, suggesting that one time cell administration is not sufficient to improve tissue recovery after stroke in the aged brain environment. Furthermore, G-CSF + BM MSCs may not impact at all on the spatial reference-memory task or infarct volume and therefore did not further improve the post-stroke recovery. We conclude that in a real clinical practice involving older post-stroke patients, successful regenerative therapies would have to be carried out for a much longer time. The BM MSC therapy warrants further investigation including repeated administrations of therapeutic cells at several time points after stroke and using various combinations with G-CSF or other relevant growth factors/cytokines. Another point that should be considered in future studies is a problem associated with autologous transplantation of BM MSCs in elderly patients, in view of an age-related decrease in the functional capacity of stem cells (reviewed by Moskalev et al., 2013).

## Conflict of Interest Statement

The authors declare that the research was conducted in the absence of any commercial or financial relationships that could be construed as a potential conflict of interest.

## Footnotes

^1^http://www.tingomat.com
